# Spatiotemporal Distribution of *Salmonella enterica* in European Hedgehogs in Northern Italy

**DOI:** 10.3390/pathogens12070946

**Published:** 2023-07-17

**Authors:** Maya Carrera, Clara Tolini, Tiziana Trogu, Andrea Boscarino, Vito Tranquillo, Martina Munari, Emanuele Callegari, Davide Tartari, Ana Moreno, Silva Rubini

**Affiliations:** 1 Istituto Zooprofilattico Sperimentale della Lombardia e dell’Emilia Romagna “Bruno Ubertini” (IZSLER), Via Antonio Bianchi 7/9, 25124 Brescia, Italy; 2 LIPU Lega Italiana per la Protezione degli Uccelli, “Il Giardino delle Capinere”, Via Porta Catena 118, 44122 Ferrara, Italy

**Keywords:** *Erinaceus europaeus*, *Salmonella* spp., Enterobacteriaceae, epidemiology, zoonosis, wildlife, Italy

## Abstract

Growing attention is being given to the European hedgehog (*Erinaceus europaeus*) because of its synanthropic behaviour and its potential role in harbouring parasites, viruses, fungi and bacteria and disseminating them to several animals and humans. *Salmonella* are the most frequently detected zoonotic bacteria that hedgehogs could transmit through contaminating water and food sources with faeces. This study aimed to determine the prevalence and distribution of *Salmonella* spp. in wild hedgehogs in the Emilia-Romagna region (northern Italy). From 2019 to 2022, 212 European hedgehogs that died naturally were tested for *Salmonella* spp. through culture isolation. Positive samples were subjected to serological typing. A total of 82 samples tested positive for *Salmonella* spp., with the overall Bayesian posterior estimated prevalence ranging from 35% (95% CI: 23–47%) to a maximum of 45% (95% CI: 31–59%) during the years considered and with an overall prevalence calculated at 39% (95% CI: 33–45%). *Salmonella enterica* Enteritidis and Veneziana were the most prevalent detected serovars in 65% and 17% of the positive samples, respectively. Since 2021, *S.* Typhimurium, *S.* Typhimurium Monofasica, *S.* Zaiman, *S.* Hessarek, *S.* Muenster, *S.* Isangi serovars, *S. enterica* subsp. Diarizonae and *S. enterica* subsp. Houtenae have been detected. These findings show a high prevalence of *Salmonella* spp. in tested hedgehogs, suggesting an important role of this animal species in the epidemiology of potentially zoonotic serovars circulating in the Emilia-Romagna region.

## 1. Introduction

The European hedgehog (*Erinaceus europaeus*) is a hibernating mammal widely distributed in Europe that is characterised by its synanthropic behaviour, which usually leads to frequent contact with humans and domestic and wild animals [1]. The hedgehog has been identified as a potential reservoir of several pathogens, such as viruses, bacteria and fungi [2]. Its interaction with several animals and its high population density make it an ideal candidate for spreading and circulating zoonotic pathogens [3,4]. In addition, hedgehogs withstand phoretic relationships with hematophagous vectors, such as ticks and ectoparasites, and can, therefore, distribute them to other mammalian species [1,5,6]. Moreover, hedgehogs may contribute to maintaining ticks in suburban areas, especially during cold months, and may play a role in the epidemiology of arthropod-borne diseases [7,8,9]. The two species of ticks that infest European hedgehogs are *Ixodes ricinus* and *I. hexagonus*, which are both vectors of *Borrelia burgdorferi* [10,11,12] and *Anaplasma phagocytophilum* [13,14], the pathogens responsible for Lyme disease and granulocytic anaplasmosis, respectively, in humans and animals. Several viruses have been found in hedgehogs, including the *tick-borne encephalitis virus* [15,16,17] and *coronavirus* [18,19,20], all of which, in addition to being potential etiological agents of disease in hedgehogs, can be transmitted to other animals [2]. Besides viruses, hedgehogs harbour different bacteria, such as *Salmonella* spp. [21], *Leptospira interrogans*, *L. ballum*, *L. borgpetersenii* [22,23,24], *Rickettsia helvetica* [25], *Mycobacterium avium* and *M. bovis* [26].

Amongst the pathogens harboured by hedgehogs, *Salmonella* spp. are some of the zoonotic bacteria of most significant concern. In fact, *Salmonella* spp. are responsible for 2.8 billion cases of human gastroenteric infections that occur annually worldwide [27], making this illness the second most commonly reported foodborne infection after campylobacteriosis [28].

The *Salmonella* species most frequently involved in animal infections is *S. enterica*, which is further divided into six subspecies, including more than 2600 serovars, distinguished by their flagellar (H) and somatic (O) structures [29]. The transmission mechanism of Salmonella is by the faecal–oral route, with the ingestion of contaminated food being the most common mode of infection [28]. Other potential transmission patterns are by human–animal contact or by indirect contact with the environment [30]. Since hedgehogs represent the most hospitalised wild animals in Italian rescue centres, workers and volunteers could be primarily exposed to hedgehog zoonotic pathogens. Therefore, rehabilitation facilities could be critical to direct human–animal transmission [4]. A study conducted in Great Britain in 2017 suggested an implication of direct human–animal contact in outbreaks occurring in the country, as a phylogenetic analysis of *Salmonella* species isolated from humans and hedgehogs showed them to be associated, suggesting that the infections may have arisen from a standard population [31]. In the United States, several cases of salmonellosis have been linked to direct contact with a pet hedgehog [32]. The hedgehog may, therefore, represent a potential reservoir of the pathogen [2,21,33]. Moreover, in pet hedgehogs, *Salmonella* infections may be asymptomatic; they can become carriers and shed the bacteria in faeces, even persistently, or they may develop a latent infection within the lymph nodes [32]. Clinical forms sometimes manifest with mucoid or bloody diarrhoea combined with enterocolitis [21]. Although there are few studies on this topic, wild hedgehogs may also exhibit this same variety of clinical symptoms. Based on the above statements, a better understanding of the role of wild hedgehogs in *Salmonella* epidemiology may yield important information from a One Health perspective [34]. In this study, we analysed 212 hedgehogs for *Salmonella* to better understand its frequency and distribution in a specific area of Emilia-Romagna.

## 2. Materials and Methods

### 2.1. Sample Collection

From 2019 to 2022, a total of 212 European hedgehogs that died naturally were collected from the wildlife recovery centre LIPU (Italian League for Birds Protection) in Ferrara (Emilia-Romagna, Italy), according to a non-probabilistic sampling method [35].

Dead animals were delivered to the diagnostic laboratory of the Istituto Zooprofilattico Sperimentale della Lombardia e dell’Emilia-Romagna, within the wildlife monitoring regional plan framework. The geographical coordinates of the locations where the animals were found were recorded, and the animals were subjected to a complete necropsy, where the contents of the large intestine were sampled and cultured for the detection of *Salmonella* spp. Thirty animals were collected in 2019 (14%), seventy-six in 2020 (36%), fifty-eight in 2021 (27%) and forty-eight in 2022 (23%).

### 2.2. Salmonella spp. Isolation and Serotyping

*Salmonella* spp. were isolated according to the ISO 6579:2002/Amd 1:2007 method (ISO 2007) [36,37]. Specifically, a pre-enrichment step was performed for the reactivation of the Salmonella spp. likely present in the test sample. This consisted of incubating 25 g of intestinal contents in sterile bags containing 225 mL of buffered peptonated water at 37 °C for 24 h. After that, a subsequent enrichment step was performed by inoculating 0.1 mL of the pre-enriched sample onto a semisolid culture medium (modified semi-solid Rappaport–Vassiliadis—MSRV; OxoidTM, Hampshire, UK) and incubating it for 48 h at 41.5 °C. Then, colonies suspected to be *Salmonella* spp. were selected by plating them on two selective solid media, xylose lysine deoxycholate agar (XLD; bioMérieux, Bagno a Ripoli, Italy) and brilliant green agar (BGA; Vacutest Kima, Arzergrande, Italy), and incubating them at 37 °C for 24 h. For confirmation testing according to the standardized method, at least one colony from a solid selective medium plate was taken. In case of negative results, four additional colonies—if available—were taken from the other combinations of selective enrichment and isolation media. Suspected colonies were plated on a Nutrient Agar plate for isolated colonies and incubated at 36 °C for 24 ± 3 h; in case of good isolation of the suspected colony, confirmation tests were performed directly, conducting Nutrient Agar seeding in parallel in order to check its purity. Pure cultures were used for subsequent biochemical and serological confirmation tests. 

Appropriate biochemical tests (Microgen^®^ GNA ID System, Microgen Bioproducts Ltd., Camberley, UK) were used to confirm the presence of *Salmonella* spp. The identification of *Salmonella* spp. serovars was conducted using a rapid slide agglutination test from pure cultures, after removing self-agglutinating serovars. An appropriate serum antigen for the detection of somatic (poly O, rabbit antiserum, SSI DIAGNOSTICS, Hillerød, Denmark) and flagellar (poly H, rabbit antiserum, SSI DIAGNOSTICS, Hillerød, Denmark) antigens was used [36].

### 2.3. Data Analysis

Considering the non-probabilistic nature of sampling in this study, a data analysis based on standard errors (such as *p*-values and confidence intervals) was not considered appropriate [38,39]. In addition, the significant/non-significant dichotomy based on a predetermined *p*-value cut-off for the interpretation of results is under reconsideration by the biomedical scientific community, as also expressed by the American Statistical Association (ASA), which has cautioned the use and interpretation of the *p*-value [40,41]. In light of this, we focused on the extent of the estimation uncertainty. 

A conjugate beta prior to the distribution of p (probability of a sample being *Salmonella*-positive) in a binomial experiment to obtain a posterior beta distribution of the probability p [42] was used to obtain point and interval estimates of the year-specific prevalence and the overall prevalence of the *Salmonella*-positive samples. Given the observed data, according to Bayes’ theorem, the posterior distribution of p is:

p|x ~ Beta (x + α, n − β)

where p is the probability of being *Salmonella*-positive; x is the number of positive samples; n is the number of tested samples; and α and β are the hyperparameters of the a priori beta distribution of p, which, in this case, was a Jeffrey’s prior with a beta distribution (0.5, 0.5). A credibility interval was then constructed from the posterior distribution, which collected the highest probability of density (HPD) corresponding to 95% of the estimated probability (p) values. The point and interval estimates of the *Salmonella* spp. prevalence were calculated using the function binom Bayes of the binom package [43] in R language [44]. The forestplot package [45] in R was used to obtain a forest plot, where all the results for each year are presented.

## 3. Results

In most cases, the hedgehogs were not suitable for a complete necropsy. Often, the carcasses were not delivered from the wildlife rescue centre immediately after death, and sometimes it was impossible to maintain the cold chain. This led to tissue deterioration and the impossibility to appreciate gross pathological lesions. 

Nevertheless, it was possible to perform a necropsy on 117 hedgehogs, 84% (n = 98) of which showed no lesions. Among the 19 remaining animals, traumatic lesions and haemorrhages were the most frequent gross pathological findings, which were probably caused by vehicle collisions and which were observed in six and four animals, respectively. Interestingly, liver necrotic foci were noticed in four hedgehogs, and in one case, they were associated with hepatomegaly. In one case, splenomegaly was observed, while in another case, jaundiced skin was evident. These six cases were the only *Salmonella*-positive animals that had gross findings. Other minor findings were pneumonia (1/19), lung congestion (1/19), ascites (1/19), enteritis (2/19) and intussusception (1/19).

**Figure 1:**
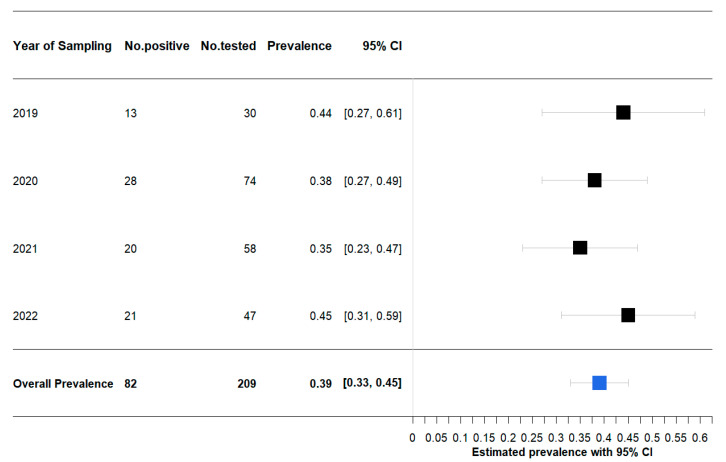
Bayesian posterior estimated prevalence with 95% credibility interval of *Salmonella* spp. isolated from tested hedgehogs.

A total of 82 samples were positive for *Salmonella*. A statistical analysis conducted over the years highlighted a rate of prevalence ranging from a minimum of 35% (95% CI: 23–47%) to a maximum of 45% (95% CI: 31–59%), with the overall prevalence calculated at 39% (95% CI: 33–45%) (Figure 1).

Three different *Salmonella* subspecies were isolated: 1.2% were *S. enterica* subsp. *diarizonae*, 1.2% were *S. enterica* subsp. *houtenae,* and 97.6% were *S. enterica* subsp. *enterica*. A total of 65% of the *S. enterica* subsp. *enterica* belonged to the serovar Enteritidis (Table 1).

The other detected serovars were Veneziana (17%), Monofasica (4%), Typhimurium (4%), Zaiman (4%), Hessarek (2%), Isangi (1%) and Muenster (1%) (Table 1).

**Figure 2:**
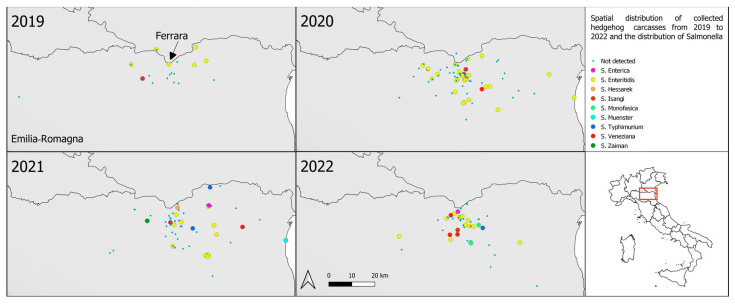
Spatial distribution of collected hedgehog carcasses from 2019 to 2022 and the distribution of the different isolated serovars of *Salmonella* spp.

Geographical coordinates were registered and used to create a map representing the location of the tested hedgehog carcasses and the eventual isolated serovars (Figure 2). The carcasses were mainly distributed in the north-eastern area of the province, and, except for isolated cases, *Salmonella*-positive hedgehogs were mainly found in the urban and peri-urban area of Ferrara and small villages.

## 4. Discussion

The current study highlighted the presence and distribution of different *Salmonella* subspecies and serovars in wild hedgehogs collected in the Emilia-Romagna region. From 2019 to 2022, 82 out of 212 tested animals were positive for *Salmonella* spp. The most frequently isolated subspecies was *S. enterica* subsp. *enterica*; within this group, Enteritidis and Veneziana were the first and second most commonly isolated serovars, respectively. These results are consistent with previous studies conducted in other European countries [31,46]. Interestingly, Enteritidis belongs to the top five *Salmonella enterica* serovars most commonly involved in human infections [28]. In addition, *Salmonella* Typhimurium, Typhimurium Monofasica, Zaiman, Hessarek, Muenster and Isangi were detected in lower numbers. Other *S. enterica* subspecies detected in our study were *S. enterica* subsp. *diarizonae* and *S. enterica* subsp. houtenae.

Several *Salmonella* spp. serovars found in our study had not yet been described in hedgehogs, as in the case of S. Veneziana, which was previously found mostly in wild boars and other wild animals in Italy [36,47,48,49]. In particular, Veneziana was the most frequently detected *S. enterica* serovar in foxes and badgers, with a prevalence of 16.4% in the same area and in a similar year range as in the present investigation [36]. The other *S. enterica* serovars detected were Hessarek, which has been described in avian species [50,51] and wild boars in Italy [52], and Zaiman, a rare serovar first detected in the Zaiman river and sporadically in humans in Argentina [53,54]. *S.* Zaiman was also isolated from foxes [34] and wild boar carcasses in Italy [55]. *S.* Veneziana, *S. enterica* subsp. *diarizonae* and *S. enterica* subsp. *houtenae* have been detected in foxes in the same area, although with a low percentage (2.99% and 1.49%, respectively) [36]. These findings show that several *Salmonella enterica* subspecies and serovars are well adapted to different wild animals and that hedgehogs may play an essential role in the dissemination of various and unusual serovars of *Salmonella*, as they could share the same environment as wild boars and carnivores.

Although only partially exhaustive, our work provides an excellent general overview of the various serovars circulating in the study area. Similar studies conducted worldwide in previous years have shown rather heterogeneous data, highlighting the need for more passive surveillance of this type of animal. Most of these studies show a similar or lower prevalence than the data presented here [5,31,56,57,58,59,60,61]. In several studies conducted in Northern European countries such as Denmark, Norway and the UK [31,58,59], the sample size was considerable, although only in one case was it higher than in our study [59]. In other studies also conducted outside Europe, the collected samples were often small and probably not representative of the real circulation of *Salmonella* in the hedgehog population [56,60,61,62,63]. In addition, it is important to note that former studies performed the analysis using a great variability in testing matrices and methods, presenting significant challenges in terms of data comparison and the characterization of *Salmonella* epidemiology in different countries.

Furthermore, in the present study, it is possible to notice an increase of different serovars over the years considered. In particular, in 2019 and 2020, the only isolated serovars were Enteritidis and Veneziana, while the others were isolated from 2021 onward. Defining this trend is clearly challenging due to the lack of available data, making it difficult to adequately explain this increase. Given the scarcity of information, further *Salmonella* spp. isolation and serotyping analysis performed on different species within the same area may provide valuable insights and potential explanations for the observed increasing trend in serovars. The rise in the number of diverse asymptomatic reservoirs could play a significant role in increasing the silent circulation of different *Salmonella* spp. serovars within the same geographical area. The ongoing monitoring of *Salmonella* spp. in wild species distributed throughout the territory will allow for a better understanding of this phenomenon [28]. 

Moreover, in our study, the finding of positive cases near urban and peri-urban areas deserves attention. Since hedgehogs are highly adaptable to urban environments [64], they also represent a threat to domestic animals and humans. As a matter of fact, some studies have shown a possible link between the contamination of farm animals and contact with wildlife [60,65,66,67]. In areas with intensive animal farming, such as the Emilia-Romagna region, farm animals may come into contact with infected animals, faeces or contaminated food and water and may in turn infect humans [67]. Therefore, hedgehogs may represent a powerful reservoir of pathogens, as they can easily access farming territories and contaminate the environment with their faeces.

Although more evidence is needed, monitoring wildlife could act as a sentinel for upcoming outbreaks in animals and humans [67]. This concept is fundamental, since in the last years, the close connection between wildlife pathogens, the environment and human health has become more and more of a point of interest. This connection can provide helpful information for zoonotic disease risk assessments, especially regarding emerging new threats [68]. In this context, investigating wildlife zoonosis, such as salmonellosis, and its relationship with spillover and transmission events is an important point to consider in many surveillance programs. In Italy, the attention given to this pathogen is very great because it was the zoonosis with the highest number of reported cases in 2021, registering a 39% increase from the previous year [28]. Furthermore, it is relevant to monitor the circulating serovars, since, according to the EFSA and ECDC, in 2021, there were 60,050 reported cases of salmonellosis all around Europe, which led to 71 deaths [28].

## 5. Conclusions

Our findings highlight the first detection of several *Salmonella* serovars in hedgehogs in Italy, with some being potentially zoonotic. The high percentage of positivity suggests that wild hedgehogs can play a role in the epidemiology and circulation of different *Salmonella* subspecies and serovars in the studied geographical area. The most commonly isolated subspecies was *S. enterica* subsp. enterica, with Enteritidis and Veneziana being the most frequently detected serovars. The increasing diversity of serovars observed over the years is challenging to explain given the scarcity of data. Examining data from different species that harbor the same pathogens within the same area could provide valuable insights into this trend. Ongoing monitoring of *Salmonella* in wild species will enhance our understanding of this phenomenon and its potential implications for animal and human health. In fact, monitoring wildlife can serve as an early warning system for identifying emerging outbreaks in animals and humans. This is especially important considering the growing recognition of the interconnectedness between wildlife pathogens, the environment, and human health. Understanding wildlife zoonoses and both spillover and transmission events is crucial for effective surveillance programs. Given the high number of reported cases of salmonellosis in Europe, including Italy, it is pivotal to monitor the circulating serovars to assess the risks of zoonotic diseases and address emerging threats.

## Figures and Tables

**Table 1:** *S. enterica* subspecies and serovars collected during the timeframe of the study, along with the overall number and percentage of different isolates.

	No. Isolates/Year				
*S. enterica* subsp. *enterica* Serovars	2019	2020	2021	2022	**Tot.**
*S.* Enteritidis	12	22	9	10	53 (65%)
*S.* Veneziana	1	6	3	4	14 (17%)
*S.* Typhimurium	0	0	2	1	3 (4%)
*S.* Monofasica	0	0	0	3	3 (4%)
*S.* Zaiman	0	0	2	1	3 (4%)
*S.* Hessarek	0	0	2	0	2 (2%)
*S.* Isangi	0	0	0	1	1 (1%)
*S.* Muenster	0	0	1	0	1 (1%)
*S. enterica* subsp. *diarizonae*	0	0	1	0	1 (1%)
*S. enterica* subsp. *houtenae*	0	0	0	1	1 (1%)

## Data Availability

The data generated or analysed during this study are included in the published article.
